# The lived experiences and explanation of women’s postpartum sexual and reproductive health: a qualitative study

**DOI:** 10.1186/s12978-025-02232-6

**Published:** 2025-12-16

**Authors:** Nazanin Rezaei, Masoumeh Namazi, Atbin Tahmasebi, Somayeh Moukhah, Zahra Behboodi Moghadam

**Affiliations:** 1 Department of Midwifery, School of Nursing and Midwifery, Ilam University of Medical Sciences, Ilam, Iran; 2 Department of Midwifery and Reproductive Health, School of Nursing and Midwifery, Tehran University of Medical Sciences, Tehran, Iran; 3 School of Medicine, Student Research Committee, Ilam University of Medical Sciences, Ilam, Iran

**Keywords:** Females, Women's, Qualitative study, Postpartum, Sexual health, Reproductive health

## Abstract

**Background:**

Despite the significance of the postpartum period, women’s postpartum sexual and reproductive health (SRH) has been neglected. This study examines women’s perceptions and experiences of SRH in the postpartum period.

**Methods:**

This qualitative study utilized conventional content analysis. The research was conducted in health centers in Ilam city, Iran. Purposive sampling was used to interview 17 mothers (service recipients) and six midwives (service providers) from November 5, 2022, to March 16, 2023. Data were collected through semi-structured interviews and analyzed using the Graneheim and Lundman approach.

**Results:**

The analysis of the interviews led to the emergence of four categories: marital dissatisfaction, lack of spousal and family support, psychological conflicts, and a defective health system, with 14 subcategories.

**Conclusion:**

The study results indicate that women’s postpartum SRH has been neglected due to a complex interplay of emotional, familial, and systemic factors. Women often lack a comprehensive and correct perception of their SRH needs in this period. Our findings necessitate a shift toward holistic care, including mandatory spousal education and improved psychological screening.

## Plain language summary

The postpartum period is associated with fundamental physical and mental changes in the mother. Many mothers and even service providers do not have enough knowledge and information about the rights and needs of sexual and reproductive health of mothers in the postpartum period. The present qualitative study examines the concept of reproductive and sexual health in the postpartum period among Iranian women.

This study shows the low quality and quantity of sexual and reproductive health care during the postpartum period and calls for improving the quality and quantity of these cares.

## Introduction

The postpartum period is a phase in which women undergo significant physiological and psychological changes after childbirth. This stage involves physical recovery, hormonal adjustments, and adaptation to the new role of motherhood. The significance of the postpartum period for mothers, newborns, parents of newborns, and their families [1] important that in some countries, the birth of a child is the ultimate goal of marriage and a symbol of femininity [2] The postpartum period is a time when spouses adapt to their parenting roles and resume their sexual activities [3]. Most mothers face many complications during pregnancy and childbirth, including vulnerability [4], physical problems [5–7], psychological problems and suicidal ideations [8, 9], and sexual problems [3, 10]; these factors eventually cause disruption in marital relations [11]. Physiological, psychological, social, and cultural changes occurring during this period all affect women’s sexual health and reproductive behavior [12, 13]. Globally, up to 90% of women report at least one sexual problem in the first three months postpartum, and the prevalence of postpartum depression is estimated to be between 10% and 15% [14, 15]. In Iran, studies have similarly shown a high incidence of sexual dysfunction, exceeding 60%, and a substantial percentage of women experiencing poor quality of life [3, 16, 17].Potential stressors for the mother in the postpartum period cause sleep deprivation, interference in relationships (personal, sexual, and social), anxiety about parenting skills, lifestyle changes, and increased need for social support [8, 18, 19], which eventually reduce the mother’s quality of life [1].

Due to the impact of unequal decision-making and societal gender roles in relationships, women in the postpartum period face significant challenges in both developing and even developed countries [8]. Still, in many developed and developing countries, most women are deprived of the necessary postpartum health and care services [4, 20].

The lack of comprehensive perception among women regarding postpartum sexual and reproductive health (SRH) is often attributed to insufficient education, cultural taboos, and the overemphasis on neonatal care, which leaves postpartum women’s SRH needs largely unaddressed. Women’s sexual health is often defined only in relation to their reproductive capacity, and in most cases, women’s sexual function or dysfunction is ignored [21]. Health systems tend to neglect *postpartum* care services related to the mother’s sexual and reproductive health (SRH) when providing reproductive health care during or before pregnancy [14, 22, 23]; healthcare staff often focus on prenatal care [18] or care related to the newborn or child [1, 18, 24]. Meanwhile, women in this period visit health centers to receive routine postpartum care for themselves and postnatal care for their child and can easily benefit from such care as well [23, 25].

Globally, postpartum sexual health is a complex issue. Recent systematic reviews and meta-analyses indicate that the prevalence of early resumption of sexual intercourse varies significantly across regions, influenced by factors such as mode of delivery and perineal trauma [26]. Studies have shown that predictors of postpartum sexual activity and function include not only physical recovery but also psychological well-being and relationship quality [27]. However, sexual health in this period remains “complex and multifaceted,” often overshadowed by the focus on the newborn [28]. In Iran, studies have shown a high incidence of sexual dysfunction and a substantial percentage of women experiencing poor quality of life [29].

Potential stressors, including sleep deprivation, interference in personal and social relationships, anxiety about parenting skills, and lifestyle changes, for the mother in the postpartum period may cause physical and psychological strain, which eventually may reduce the mother’s quality of life [1, 18, 19]. Due to the impact of unequal decision-making and societal gender roles, women in the postpartum period face significant challenges in both low-income and high-income countries [8].

Although SRH care has generally improved through measures such as increased access to healthcare services and policy reforms, and while these efforts have enhanced awareness and accessibility, we are still far from the initial target, especially with regard to women’s postpartum SRH [30–32].

In the specific context of Iran, women’s sexual and reproductive health is deeply influenced by cultural and religious norms. Sexual health is often considered a taboo subject, and discussion about sexual dysfunction is frequently stigmatized. The cultural expectation of the “sacrificing mother” prioritizes the child’s needs above the mother’s well-being. Additionally, the patriarchal structure in some families may limit women’s autonomy in seeking SRH care. These cultural factors, combined with a healthcare system that traditionally focuses on physical rather than holistic post-natal recovery, create unique barriers for Iranian women in expressing their SRH needs.

Collecting data related to mothers’ physical, sexual, and psychological complications in the postpartum period can help promote their postpartum SRH [23]. Given the complex, sensitive, and multi-dimensional nature of postpartum SRH, and the necessity of deeply exploring the underlying perceptions and contextual factors that quantitative methodologies often overlook, the present study was conducted to explain women’s perceptions and experiences about their postpartum SRH.

## Materials and methods

This qualitative study was designed based on the Consolidated Criteria for Reporting Qualitative Research (COREQ) [33].

### Study design and subjects

This is the qualitative part of a mixed-methods research conducted using conventional content analysis.

### Data collection and participants

The study was conducted in health centers across five different districts of Ilam city (North, South, East, West, and Central) to ensure diversity in the setting selection. Purposive sampling with maximum variation in age, parity, and postpartum time was utilized to recruit participants. The participants included 17 eligible mothers (service recipients) and six midwives (service providers). Data collection took place from November 5, 2022, to March 16, 2023, and continued until data saturation was achieved, which was determined when no new codes, themes, or information emerged from the subsequent interviews.

The inclusion criteria for the mothers were: being in the postpartum period (six weeks to 12 months after childbirth), being literate, having only one living healthy child (to minimize the confounding effect of multi-parity), no experience of major stressful life events over the past six months (e.g., divorce, death of a first-degree relative) to differentiate postpartum-specific stress from external situational crises, and no childbirth complications leading to hospitalization. The inclusion criteria for the service providers (midwives) were: having at least 5 years of work experience in postpartum care units and willingness to share their experiences regarding challenges in providing SRH care.

After explaining the study purpose and obtaining informed consent, semi-structured, in-depth, face-to-face interviews were conducted. The interviews took place in a private environment at health care centers or a location preferred by the participant to ensure privacy. All interviews were conducted by a female researcher in the local languages (Kurdish and Luri) to ensure participants’ comfort and accurate data collection. To ensure reflexivity, the interviewer (N.R.) is a female faculty member in Midwifery with extensive experience in maternity hospitals. Her background allowed for building rapport while maintaining professional boundaries, and the research team engaged in peer debriefing to minimize bias.

With the participants’ permission, the interviews were audio-recorded. The interview process began with general open-ended questions to establish rapport, followed by specific questions regarding the research objectives, and continued with probing questions.

Examples of questions asked to the service recipients (mothers) included:

- What changes do you feel in your sexual relationship with your husband in the postpartum period?
- What do you think about your husband’s and your family’s support in this period?
- How do you feel about the changes in your body?

Examples of questions asked to the service providers (midwives) included:

- What are the main sexual and reproductive health concerns reported by mothers?
- What challenges do you face in providing SRH counseling to postpartum women?
- How do you perceive the role of spouses in postpartum care visits?

### Data analysis

The interviews were carefully typed verbatim on the same day of the interview and then coded. In this type of study, themes do not exist in advance and are extracted from information. Each subsequent interview was conducted after the previous one was analyzed. Data analysis was performed by Zhang & Wildemuth’s method:

- Preparing the data for qualitative content analysis: The recorded interviews were converted into text format.
- Defining the unit of analysis: Each transcript was entered into the qualitative data analysis software as a unit of analysis; then, the transcripts were coded by identifying the meaningful units.
- Categorization and coding: Categories were extracted from the data inductively with constant comparison.
- Testing the coding plan in the text sample: Coding was performed in a sample of the text; then, to ensure the stability of the coding, the data were controlled by two other research team members.
- Coding the entire text: After the research team reached consensus regarding the stability of the coding, the coding process was applied to the entire text.
- Re-examining the codes regarding coding stability: The primary codes, sub-categories, and categories were rechecked by two research team members and those experienced in qualitative research to prevent human error.
- Drawing conclusions from the coded and categorized data: This step involved identifying the features and dimensions of the categories, the relationships between the categories, discovering the patterns, and testing the codes against the full range of the data.
- Finally, to ensure the study’s replicability, the categories were reported [34–37].

### Trustworthiness

The credibility, dependability, confirmability, and transferability of data were checked using Lincoln and Guba’s criteria [38]. To ensure dependability of the data, two research team members carefully read the interview transcripts and performed coding, categorization, and review. For credibility, the researcher engaged with the participants through semi-structured in-depth interviews and constant review of the interview notes and data. The confirmability of the data was checked by giving the extracted codes to the participants. To ensure data transferability, purposive interviews were held with maximum variation, and the research procedure and a sample of participants’ statements were presented in full.

## Results

In the analysis of the transcripts, 14 subcategories and four categories emerged. The total number of interviews was 23 (six with midwives and 17 with mothers). All the mothers reported that their pregnancy was planned. Four of them reported that they had not received any support during their postpartum period. Six mothers had one source of support, and seven mothers had all three sources of support (husband, own family, and in-laws). Thirteen mothers were housewives and four were employed. The mean duration of each interview was 57.92 min. Additional demographic data pertinent to the study participants are summarized in (Table 1).

**Table 1:** Demographic characteristics of the participants of the individual interviews

Variable	Number	Mean age	Level of education	Type of childbirth	Time elapsed since childbirth (months)
Service providers (midwives)	6	44.52 ± 8.9	Bachelor’s degree, *n* = 3	-	-
			Master’s degree, *n* = 3		
Service recipients (mothers)	17	4.00 ± 30.94	Master’s degree or higher, *n* = 5	9 = NVD 8 = C/S	1.5–3 months: *n* = 4
			Bachelor’s degree, *n* = 6		3–6 months: *n* = 4
			Below high school diploma, *n* = 1		6–9 months: *n* = 5
					9–12 months: *n* = 4

*NVD* Normal Vaginal Delivery, *C/S* Cesarean Section

The results of the conventional content analysis led to four categories and 14 subcategories. These categories are summarized in Table 2.

**Table 2:** The results of the conventional content analysis

Categories	Subcategories	Description of Subcategory
Marital dissatisfaction	Fear	Participants’ anxiety regarding resumption of intercourse due to pain, vaginal dryness, or fear of wound dehiscence.
	Distorted body image	Negative perception of physical appearance (weight gain, skin changes) affecting self-esteem and sexual confidence.
	Fatigue	Physical exhaustion and sleep deprivation reducing sexual desire and prioritizing rest over intimacy.
	Worrying about husband’s extramarital affairs	Fear that the husband might seek sexual satisfaction outside the marriage due to the wife’s reduced availability or attractiveness.
Lack of Spousal and Family Support	Lack of support from the husband	The husband’s failure to assist with childcare or household chores, leaving the mother feeling isolated.
	Men’s lack of participation in counseling	The systematic exclusion of men from postpartum education and their reluctance to attend training sessions.
	Impact of Family Involvement	Challenges arising from either a total lack of help from extended family or intrusive interference in child-rearing.
Psychological conflicts	Worrying about the child’s health	Excessive anxiety regarding the infant’s safety, choking, or illness.
	Worrying about the child’s future	Concerns about the long-term well-being of the child, economic stability, and parental mortality.
	Ambivalence towards the child’s birth	Conflicting feelings of joy about motherhood mixed with a sense of lost freedom or regret.
	Worrying about personal health	Fears regarding permanent physical damage, weakness, or undiagnosed postpartum complications.
Defective health system	Lack of proper counseling	Insufficient knowledge or confidence among staff to address sexual health questions; superficial care.
	Non-standard physical space	Lack of privacy in clinics (e.g., shared rooms) preventing mothers from discussing sensitive sexual issues.
	Inadequate number of service providers	Shortage of staff and lack of male health workers or psychologists to support the family unit.

### Marital dissatisfaction

This category has four subcategories: Fear, distorted body image, fatigue, and worrying about the husband's extramarital affairs.

#### Fear

Due to the physical and psychological changes occurring in women during pregnancy and after childbirth, some women are afraid of having sex at this time due to possible complications:

- A mother (nine months after childbirth) said: “My body felt dry. I was afraid because of this dry sensation in my body. I didn’t like to have sex before two months had passed since my childbirth because I was afraid the stitches would open up” (participant 5).
- Another mother (five months after childbirth) said: “I was afraid of intercourse because of my bleeding. I was afraid that if I had sex, my bleeding would get worse, or that my uterus would rupture due to bleeding and I’d get hurt” (participant 11).

#### Distorted body image

Some changes occurring during pregnancy take a long time to return to their original state after childbirth, or they rarely do. These changes, which include skin discoloration (face, genitals, and breasts), weight gain, hair loss, or sagging breasts, cause worries for many mothers or reduce the quality of sex for many couples.

- A mother who had a five-month-old infant said, "I feel that my body is not stitched up properly and its shape is distorted and looks all weird. I don't like my vagina myself. My breasts are sagging, and I don't like them either" (participant 15).
- Another mother (seven months after childbirth) said worriedly: "I’m very upset that my body has become dark and I've gotten fat. I don't feel alright. I'm always browsing [social media] channels that discuss how you can lose weight. My husband tells me to do something for myself because I'm a mess" (participant 7).

#### Fatigue

Having gone through pregnancy and childbirth as well as the restlessness and excessive crying of the infant in the first weeks cause excessive fatigue in the mother, which can reduce sexual desire and affect the sexual relationship of the couple:

- A mother with a seven-month-old infant noted: "I distance myself a lot from my husband because I'm tired and have insomnia. I think everyone likes sex. It's a lie to say I don't like it. My husband wants it too, but I tell him I'm tired" (participant 7).
- Another mother with a five-month-old infant stated: "I don't think a mother hates having sex, but what can she do? Fatigue makes people assign the least priority to these things. Sometimes my husband gets nervous and disappointed because we haven’t had sex for a while" (participant 17).

#### Worrying about the husband's extramarital affairs

Due to the physical and psychological changes during pregnancy and after childbirth, the sexual relationship of the couple sometimes gets disturbed. This issue makes some men seek to satisfy their unfulfilled sexual needs during pregnancy and after childbirth through extramarital affairs. Although most women do not express this concern, they always feel its possibility:

- A service provider with about 30 years of experience in service provision to mothers said: "I remember that a patient had come to the clinic once. According to her, her husband said that her body had become loose after childbirth and wasn't like it was before, so he didn't feel the way he used to about it. She said she was afraid he might sleep with someone else" (participant 23).
- Another employee of the health centers with 15 years of work experience believed that: "The conditions of a mother have changed a lot in the postpartum period compared to before pregnancy; one must understand when this woman can be mentally and physically ready to have sex. Sometimes, the husband doesn't understand the mother well and goes after extramarital affairs" (participant 21).

### Lack of spousal and family support

This category has three sub-categories: Lack of support from the husband, men's lack of participation in counseling and training sessions, and having the support of the family.

#### Lack of support from the husband

The mother becomes very fragile and irritable during this period due to experiencing problems related to the postpartum period, such as bleeding, pain, insomnia, fatigue, and worrying for the infant. Not having the support of the husband during this period can aggravate her problems. Nonetheless, mothers who benefit from the support of close people, especially their husband, during this period feel more capable of tolerating and adapting to the stress and hardships of this period:

- A mother with a three-month-old infant noted: "I was completely alone for about ten days. I had no one to take care of me. My husband was at work until the evening, slept in a separate room at night and closed the door so that the baby's crying wouldn't bother him" (participant 6).
- Another mother with an 11-month-old infant said: "My husband is a bank employee; he should focus all his attention on his job so that he makes no mistake. I have to do everything by myself. Sometimes, he says, 'Well, you're the mother; it's the mother’s duty!'" (participant 8).

#### Men's lack of participation in counseling and training sessions

Men's participation in various areas of SRH can strengthen relationships within the family and increase the sense of responsibility in men. Nonetheless, despite the importance of their participation, their place in important life events, including the postpartum period, is still not well discussed:

- An employee with 27 years of experience providing services in health centers believed: "The husband is abandoned during pregnancy and after childbirth and is not involved in any training anywhere. There's no role mentioned for the husband in maintaining and improving the family's reproductive health. Our main problem is the husband! Husbands either don't visit or don't believe in training" (participant 22).
- A mother whose child was three months old said: “When I visit the health center, I only go to get the baby vaccinated, not to get care for myself. Because my husband wants to go to work afterwards, he doesn't let me ask any questions about myself. He insists that I hurry up, get the baby vaccinated and leave. When he gets angry, it gets out of control. So, I say, ‘OK, let's go back” (participant 6).

#### Having the support of the family

Having family support in the postpartum period can help the mother both physically and psychologically and also effectively reduce her physical and psychological illness:

- One of the service providers said: "The mother is supported by many family members when she is pregnant, but as soon as she gives birth, the family’s entire attention goes to the infant, and the mother gets abandoned" (participant 22).
- A mother whose infant was two months old and who complained about the lack of support during this time noted: "I had no support at all, neither from my family nor my in-laws. Because I don't sleep well at night, I'm really suffering. I'm having a hard time –a very hard time!" (participant 1).

### Psychological conflicts

This category emerged with four subcategories: Worrying about the child's health, worrying about the child's future, ambivalence towards the child's birth, and worrying about personal health.

#### Worrying about the child's health

Worrying about the child's health is the first subcategory of psychological conflicts. Mothers’ worries are considered normal in some cases, but sometimes, they get abnormally frightened and anxious about their infant's health:

- A mother who had a three-month-old infant said: "I heard that some infants get choked to death. I have sometimes breastfed the child and burped him too, but then, he threw up again and might have choked if I weren't nearby" (participant 16).
- Another mother, who was a midwife and whose baby was 11 months old, said: "My biggest concern right now is that I'm not with the baby. Although I trust the nanny, I’m more worried about the baby; what if something gets stuck in his throat and the nanny can't do anything about it?” (participant 8).

#### Worrying about the child's future

Since becoming a mother, women often consider their child and everything about them as the most important concern in their life. Some get worried about their child's future due to unfavorable and unstable economic conditions or for other reasons:

- A mother with a three-month-old infant noted: "Now I'm mostly concerned about my child. I don't want my child to experience the hardships that I went through. God gave me this child, but now I'm responsible for him. This sense of responsibility towards the child is very consuming" (participant 6).
- A mother who had a four-month-old child said: "Sometimes, I worry a little. Sometimes, I wonder what happens if I'm not there, how will my child grow up?" (participant 2).

#### Ambivalence towards the child’s birth

During the postpartum period, although the mother is experiencing the joy of motherhood, she might occasionally feel psychological conflicts and ambivalence, especially if it is her first experience of motherhood, and she might therefore develop concerns about the baby’s future in addition to the happiness she feels:

- A mother who had given birth three months before said: "I worry sometimes. I think that becoming a mother is a positive emotion given by God. Before becoming a mother, I wasn't ready to give up my sleep for anyone or anything, but now, when I'm woken up, I'm happy and not angry. You know, the arrival of the child has given my life a purpose. It's like a dirt road that was messy, and now, there's asphalt pavement with a clean and tidy end" (participant 14).
- Another mother whose infant was three months old said: "Becoming a mother is a very good feeling, a very special sense. You feel a big change has happened in your life. But at first, I was worried and saw the birth of our child as a bad thing. It was as if my freedom was taken away from me” (participant 6).

#### Worrying about personal health

Physical and psychological changes during pregnancy and after childbirth lead to worries about personal health disorders in the mother. At this time, the mother is worried that she may not be able to regain her lost energy or that the problems created will be permanent:

- A mother who had given birth nine months before and complained deeply about the poor quality of postpartum care said: "I don't want to have children anymore because I'm afraid that the problems I faced after this delivery will happen again. When I was discharged after giving birth and came home, I had problems urinating, and I was in pain until the morning. I could've died, but no one believed me!" (participant 5).
- A mother who had a five-month-old infant said: "One of the midwives told me that you have varicose veins on your legs. This worried me a lot. I’m worried that something might happen to me. Overall, pregnancy, childbirth, and child care harm the body way too much. How should I put it? The body becomes weak and may suffer severe complications later!" (participant 5).

### Defective health system

This category has three sub-categories: The lack of proper counseling by trained personnel, non-standard physical space, and inadequate number of healthcare service providers.

#### The lack of proper counseling by trained personnel

Due to the changes in the job title of midwives and other healthcare workers in the Ministry of Health and Medical Education, where all of them are now called *healthcare workers*, many caregivers who provide services to clients do not have sufficient information about the subject due to their having multiple professions or not having the time or required expertise about the services they provide:

- A mother whose child was eight months old said: "I'd like them to give me info about how to have sex or use methods of contraception or disease prevention. But no one provides such training. They don't spend enough time with the mother during this period. Sometimes, they just ask a general question, like 'Do you have any marital problems?' If I say no, the doctor or midwife won't ask me anything else" (participant 13).
- A healthcare worker with 15 years of experience said, "I think it would be very good for us as midwives to get some training on communication with the clients, especially with regards to sexual problems and so on. We have issues with this subject. We don't know what's right and what's wrong. Sometimes I don't know the answer to a mother's questions or don't have the confidence to answer them" (participant 19).

#### Non-standard physical space

In order to provide SRH-related care, it is necessary to have a suitable physical space so that mothers and families can easily use these services:

- A service provider with 15 years of experience said: "Due to our culture and lack of ability to respect privacy, the mother or her husband can’t easily tell us about their sexual problems. For example, I had a client whose husband only had anal sex with her, and although the woman was upset about this, it took her a long time to tell me about it with great difficulty" (participant 22).
- A service provider noted: "To be honest, we don't ask in detail about the mother's problems. We share the hallway in which we work, and everyone's listening. Even if we ask, the mother won't tell the truth in this situation" (participant 18).

#### Inadequate number of service providers

Another crucial factor in providing quality SRH services is having an adequate number of service providers for the family and clients. When the number of caregivers is not proportional to the number of clients, the quality of care decreases:

- A service provider with 28 years of experience said: "Sometimes, a woman's husband does not visit because he doesn't like to be trained by a woman or because the midwife doesn't give him proper training. The truth is that it is a bit difficult to communicate with a man; it requires skills, knowledge, the presence of a male healthcare worker in the center, and also enough time. All healthcare workers are women right now, and this makes it really difficult” (participant 18).
- A healthcare worker with 18 years of experience believed, "I think all mothers who are apparently healthy should also be screened psychologically. Sometimes, we examined a mother based on the questions on the website, and the mother was evaluated as healthy, but later, the same mother committed suicide. We don't have any space for providing psychological counseling here. They've given this room to a doctor, who doesn't do anything at all really!" (participant 19).

## Discussion

The results of the present study provided a deep understanding of the complex challenges and lived experiences that women face in relation to their postpartum Sexual and Reproductive Health (SRH). The findings suggest that women’s SRH is often undermined by a complex interplay of personal fears, marital dynamics, inadequate family support, and systemic failures within the healthcare system.

 Marital Dissatisfaction as the Primary Barrier One of the most important problems reported by the women in this study is marital dissatisfaction, characterized by fear, distorted body image, and fatigue. Fear emerged as the most prominent cause, with participants expressing deep anxiety over vaginal dryness and the concern that stitches would open up. This finding is consistent with previous studies by Rathfisch et al. [39] and Pardell-Dominguez et al. [40], which highlighted that physical trauma and pain are significant barriers to resuming sexual activity. However, our qualitative data reveal that this fear is not merely physical but is exacerbated by a lack of explicit information from healthcare providers. This aligns with the scoping review by Wood et al. [21], which emphasized that insufficient sexual counseling leads to the proliferation of myths and unnecessary anxiety.

Similarly, distorted body image was a major contributor to reduced marital satisfaction. Consistent with Rahmani et al. [29], our participants reported that changes such as weight gain and skin discoloration negatively affected their sexual quality of life. However, unlike quantitative studies such as Kerrigan et al. [41], which primarily focus on the clinical correlation between obesity and health outcomes, our findings underscore the psychological impact of the husband’s negative perception. Our participants indicated that the husband’s criticism acts as a direct driver of distress and fear of extramarital affairs, adding a relational dimension to the issue of body image.

 The Central Role of Lack of Spousal and Family Support The second category, Lack of Spousal and Family Support, highlights that increased maternal responsibilities often lead to extreme fatigue and a pervasive feeling of abandonment. This finding corroborates the work of Razurel et al. [4], who identified social support as a crucial buffer against postpartum stress. In contrast, however, our data reveal a significant systemic failure regarding men’s lack of participation in counseling. While studies by Davis et al. [42] and van Vulpen et al. [43] have demonstrated the benefits of male involvement in reproductive health, our findings show a gap in implementation, where men are often “abandoned by the system” or do not believe in training. This contrasts with the successful joint decision-making models reported by Bunda and Amani [44] in Tanzania, suggesting that in our context, cultural norms and the lack of male healthcare workers create specific barriers to spousal inclusion.

 Psychological Conflicts and Systemic Deficiencies Psychological conflicts, such as worrying about the child’s health and future, were prevalent among participants. These anxieties echo the findings of Verreault et al. [9] regarding the high prevalence of postpartum mental distress. However, our study suggests that these personal worries are compounded by a “Defective Health System.” The participants’ reports of non-standard physical spaces and lack of privacy are similar to the challenges in low-resource settings described by Wood et al. [21]. Furthermore, consistent with the critical review by Cheng et al. [23], we found that the healthcare system tends to prioritize the newborn over the mother, leaving women feeling neglected. Unlike comprehensive care models that integrate psychological screening, our findings highlight a dangerous gap where suicide screening and psychological counseling are absent, representing a profound system deficiency that requires immediate intervention.

## Limitations

This study was conducted on postpartum women in Iran. One limitation of this study is that factors such as the lactational status and related hormonal influences were not specifically assessed, as the study focused solely on the inductively derived categories and subcategories emerging from participants’ narratives through a conventional content analysis approach.

The results of This study can be used for comparison purposes, but the findings may not be generalizable to other regions due to the unique cultural conditions and their impact on the subject.

## Conclusion

The study successfully explained the complex challenges affecting women’s postpartum SRH, which are categorized into marital dissatisfaction, lack of spousal and family support, psychological conflicts, and a defective health system. Our findings indicate that women’s postpartum SRH is deeply compromised due to a confluence of personal fears (such as fear of intercourse and body image concerns), severe spousal neglect, and systemic failures in providing sensitive and comprehensive care. Specifically, women lack a correct perception of this period’s SRH needs, and the support system is perceived as inadequate or absent. The emergent categories highlight the urgent need for a paradigm shift from a purely physical check-up model to a holistic care framework that includes mandatory spousal education, culturally sensitive care spaces that ensure privacy, and confidential psychological screening in health centers. These targeted interventions are essential to improve the quality of life and sexual well-being of mothers.

## Data Availability

The data used in this study are included in the manuscript.The data collected in this study were included in this manuscript. The data have not been stored in a publicly accessible repository. The data sets used in the analysis that support the conclusions of this study can be obtained from the corresponding author upon a reasonable request. Of course, after obtaining permission from the Ethics Committee of Tehran University of Medical Sciences.

## Abbreviations

PTSD: Posttraumatic Stress Disorder
SRH: Sexual and Reproductive Health
NICU: Neonatal Intensive Care Unit
